# Argument ordering in simple sentences is affected by age of first language acquisition: Evidence from late first language signers of ASL

**DOI:** 10.1017/S0305000924000400

**Published:** 2024-11-08

**Authors:** Rachel Miles, Marla Hatrak, Deniz İlkbaşaran, Rachel Mayberry

**Affiliations:** Department of Linguistics, University of California San Diego, La Jolla, CA, USA

**Keywords:** American Sign Language, language deprivation, word order, age of acquisition

## Abstract

Research on the language acquisition of deaf individuals who are exposed to accessible linguistic input at a variety of ages has provided evidence for a sensitive period of first language acquisition. Recent studies have shown that deaf individuals who first learn language after early childhood, late first-language learners (LL1), do not comprehend reversible Subject-Verb-Object (SVO) sentences. The present study analyzed 478 signed productions elicited with pictures depicting simple events with one or two arguments by 28 signers. The argument order patterns of native signers converged with one another and the word order patterns of American Sign Language (ASL). By contrast, the ordering patterns of the LL1 signers did not converge with one another or with the patterns of the native signers. This indicates that early childhood is a period of heightened sensitivity to basic word order and may help explain why complex structures are difficult for LL1 signers to learn.

## Introduction

Children develop language in a predictable sequence from single words and two-word combinations to simple sentences and, eventually, complex sentences. Children’s initial productions are declarative statements about events occurring in the present environment and time (Bloom et al., 1975; Brown, 1973; Vasilyeva et al., 2008). All languages have ways of encoding events by specifying the participants and their roles so that users of the language are clear as to who or what is doing what to whom or what. Children’s early productions in language tend to use the word order of the language they are exposed to. Eventually, children learn the rules for basic clause structure, specifically how to differentiate arguments in a sentence with a single verb and two arguments, S(subject) and O(object). Learning complex sentence structures – that is, structures with multiple verbs and their arguments – develops out of the acquisition of basic clause structure (Diessel, 2004; Vasilyeva et al., 2008).

While this developmental sequence appears to be universal when children have perceptual access to the language around them, it is also known that the age of language acquisition affects eventual proficiency (Lenneberg, 1967). Research has found that age of acquisition has long term effects on the acquisition of second language syntax and that these effects are modality free because they appear in the acquisition of sign language syntax as well (Boudreault & Mayberry, 2006; Cormier et al., 2012; Emmorey et al., 1995; Malaia et al., 2020; Mayberry, 1993; Newport, 1990; Novogrodsky et al., 2017). This line of research has led to the finding that the age of first as compared to second language acquisition is especially sensitive to age of language acquisition (see Mayberry & Kluender (2018) for an overview). These effects are apparent in children born severely and profoundly deaf who are at high risk for limited language experience because they cannot perceive the auditory signal of the languages spoken around them. Although they can access visual language through the visual modality, they first experience sign language at a variety of ages after infancy depending on the languages used in the family, in interventions, and school placement (Humphries et al., 2016).

Case studies of individuals born deaf who first experienced sign language long after infancy have found that they quickly learn vocabulary and produce signs and sign combinations, regardless of whether their initial language experience began in early childhood (Berk, 2003) or adolescence (Ferjan Ramírez et al., 2013). However, while the young children progressed to a two word stage in which their utterances predominantly followed the canonical word order of ASL (Lillo-Martin & Berk, 2003), the adolescent learners did not. After five years of ASL immersion, fewer than 12% of their spontaneous utterances included two arguments – that is, both subject (S) and object (O) (Cheng & Mayberry, 2018) – suggesting that the development of basic sentence structure is impacted when language acquisition is postponed until after early childhood. This hypothesis was confirmed in two studies. Signers who first learned ASL at the age of nine or older show limited comprehension of SVO sentences (Mayberry et al., 2023) and use semantic and real world cues rather than word order to comprehend reversible SVO sentences (Cheng & Mayberry, 2020).

It is possible, however, that late L1(LL1) learners have learned the SVO structure but that the tasks used in previous studies failed to reveal it. Successful performance on the previous experimental tasks required working memory which may have reduced accuracy and hence masked SVO rule learning. To test the hypothesis that learning language after early childhood affects the acquisition of basic clause structure, we employed a picture description task that requires no working memory. If the production patterns of LL1 signers diverge from those of signers who learned ASL from birth, this would confirm that age of L1 acquisition affects the development of basic clause structure. Alternatively, if age of L1 experience shows no effects on the production of argument ordering patterns, this would suggest that the critical or sensitive period primarily affects the acquisition of complex structures with basic clause structure being unaffected. To test the hypothesis, we focus on single events with one and two arguments. Before explaining the study design, we describe word order in simple intransitive and transitive ASL sentences. We relate these structures to similar ones found in other sign languages in the discussion after describing the results.

## Argument Marking in ASL

ASL uses both word order and morphological marking of arguments to indicate subject and object. Morphological marking of arguments is limited to one class of verbs, known as agreeing or indicating verbs (Padden, 1988). In morphological marking on agreement verbs, the movement direction or palm orientation of the verb indicates the subject- and object-hood of the nouns; in this case, word order can deviate from the canonical SVO order used in ASL. ASL additionally marks arguments through the use of word order. For the class of plain verbs (Padden, 1988), which cannot be modified with morphological marking described above, arguments are primarily indicated via word order. Sign languages, like the majority of spoken languages, are classified as having basic word order of either SVO or SOV (Napoli & Sutton-Spence, 2014). ASL uses the basic word order of SVO (Coons, 2022; Fischer, 1975; Liddell, 2003). In example (1), taken from Fischer (1975), arguments are disambiguated by their order of occurrence in the sentence. The man is the subject because this sign occurs in the syntactic location of the subject while the child is in the syntactic position of object.

**Table:** 

(1)	MAN	NOTICE	CHILD	(Fischer, 1975, p. 5)
	‘The man noticed the child.’			

In ASL, the basic word order of SVO is the only word order allowed in sentences with reversible arguments in the absence of explicit grammatical marking (Fischer, 1975). In addition to the morphological marking of arguments on agreeing verbs described above, alternative word orders of O,SV and OV,S (comma indicates a prosodic break) are formed through topicalization (Liddell, 1980). Grammatical non-manual marking of word order variations include prosodic breaks, grammatical facial expressions, head tilts or shakes, and shoulder or body movements. Topicalization allows for fronting of the object or verb phrase with nonmanual marking of the raised arguments.

Thus, the use of SVO to indicate which argument is the Subject and which is the Object in dual argument reversible structures provides evidence of abstract rule learning, the gateway to learning complex sentence structure.

## Childhood Acquisition of Word Order

Young children typically acquire the language of their environment, ultimately matching adult-like use of grammatical structures including argument marking. Preferential looking studies have shown that children are sensitive to basic word order of their native language in comprehension as young as 17 months (Naigles et al., 1993).Their production of basic word order occurs as soon as they utter full sentences. This is true for hearing children learning spoken language (Braine, 1963; Bloom & Capatides, 1993; Tomasello, 1992), and importantly for the present study, for deaf children learning sign language (Coerts, 2000; Pichler, 2001; Pizzio, 2013).

As described above, basic word order is a key building block in the later acquisition of more complex syntactic forms (Diessel, 2004; Slobin & Bever, 1982). It has been argued that children’s acquisition of word order is facilitated by pragmatic and semantic factors, such as animacy and real-world knowledge. Theories impute different underlying mechanisms by which children transition from their initial understanding of individual word meanings associated with concrete objects to the use of abstract word order and morphosyntactic marking. But most theories posit that linguistic input is mapped onto the basic categories of syntactic structure through language experience (Bates & MacWhinney, 1982; Pinker, 1996; Tomasello, 2003). The competition model (Bates & MacWhinney, 1989) highlights the fact that, in most cases, the word order and morphosyntactic marking of arguments is redundant with semantic and pragmatic cues. For example, in sentences such as the ‘the boy kicks the ball,’ knowledge about the real world and whether animate or inanimate things tend to be kickers can result in comprehension without reliance on word order or morphosyntactic markers. These cues remain available to adults. In fact, when word order and morphological marking of arguments is artificially stripped from sentences in an experimental setting, adults rely on such cues and can correctly interpret the meaning of most event types (Mahowald et al., 2022). The fact that in many instances word order and morphosyntactic marking are redundant with semantic and pragmatic cues can facilitate the mapping of these cues onto the word order and morphosyntax rules of a language.

But semantic and pragmatic cues can only take the language user so far because they are unavailable in reversible SVO sentences such as ‘the boy hits the girl.’ The only way to know which argument, the boy or the girl, did the act, the hitting, is to know the word order rule. In this way, the comprehension and production of sentences with two reversible arguments provides stronger evidence for abstract rule learning than sentences with irreversible arguments.

Some studies use scenarios where semantic or pragmatic cues conflict with word order and morphosyntactic marking to determine which cues the children rely more on when dealing with two argument structures. Young children have been shown to rely more on event knowledge (Chan et al., 2009; Strohner & Nelson, 1974), discourse context (Hargrove & Panagos, 1982), and animacy (Chapman & Kohn, 1978; Dodson & Tomasello, 1998) than on the morphosyntactic or word order markings of their language in these comprehension paradigms. However, by age six or seven, children who have experienced language from birth no longer depend on these cues and instead produce sentences with grammatical word order (Braine, 1963; Bloom & Capatides, 1993; Tomasello, 1992; Coerts, 2000; Pichler, 2001; Pizzio, 2013) and use morphosyntactic and word order cues in comprehension (Chan et al., 2009; Chapman & Kohn, 1978; Dodson & Tomasello, 1998; Hargrove & Panagos, 1982; Strohner & Nelson, 1974).

## Current Study

Previous studies of early word combinations produced by late first language learners provide evidence that these individuals are developing sentence structure (Berk & Lillo-Martin, 2012; Ferjan Ramírez et al., 2013; Morford, 2003). However, recent experiments suggest that the acquisition of two argument structures in ASL may be limited when language acquisition begins after early childhood (Mayberry et al., 2023). To test this hypothesis, we examined the sign language productions of eleven LL1 signers of ASL in response to pictured line drawings of three types of events: intransitive, irreversible transitive, and reversible transitive events. We predict that LL1 signers would master single argument clauses prior to dual argument clauses. For dual argument clauses, then, if the LL1 signers have mastered the syntactic word order rule, they should use it for both irreversible and reversible events. And if LL1 signers have acquired basic word order in ASL, then their argument ordering should pattern with that of the native signers. Alternatively, if the late L1 learners have not learned basic ASL clausal structure, then they should not pattern with the native signers. This would suggest that there is a sensitive or critical period for the learning of basic clause structure ending after early childhood.

## Methods

The data analyzed for the present study were obtained from an elicited production task that was part of a larger study designed to determine which ASL syntactic structures are acquired by LL1 signers. The other experiments included sentence-picture matching and elicited imitation.

### Participants

Twenty-eight adults (Table 1) who were born severely to profoundly deaf from birth participated in the study. Testing the hypothesis required recruiting participants whose early childhood was demonstrably characterized by limited perceptual access to spoken language because they could not hear, and sign language was absent from the environment. The recruited target group consisted of individuals who had only minimal experience with perceptually accessible natural language throughout early childhood, until the age of 9 years or older. The control group consisted of signers whose experience with sign language began at birth. The inclusion criterion was early versus late age of ASL immersion, birth versus age nine or older. For this reason, the groups were not matched in any way

#### Late first language signers (LL1).

Each LL1 participant was raised until the age of nine or older in an environment of limited social interaction through natural language. The seven women and four men were not known to have had auditory access to spoken language due to severe to profound hearing loss reported to have had a prelingual onset. Each LL1 participant was the only deaf member of hearing families who neither knew nor used any sign language. Nine participants were born outside the USA where they received little or no special services or education. Language immersion for these participants began when they immigrated to the USA and enrolled in schools for deaf children where ASL was used (seven participants) or in special classes with deaf peers (two participants). All but two of the LL1 participants reported communicating with their families with gesture prior to ASL immersion. One participant’s adoptive family used ASL. Another participant attended school sporadically until the age of 13. Age of ASL immersion ranged from 9 to 21 years of age with a mean of 14.5 years (SD = 3.47). Length of exposure to ASL at the time of testing ranged from 8 to 35 years with a mean of 19.8 years (SD = 8.96). The LL1 participants knew little or no spoken language. They were able to read single English words and phrases (Table 1). In another study, these and additional participants with similar backgrounds showed quantity and magnitude estimation abilities comparable to those of signers with language experience from birth indicating that lack of exposure to accessible language in early childhood does not affect all cognitive domains (Semushina & Mayberry, 2022). Most standardized cognitive measures have been normed for children with normal hearing who have had continual access to language since birth and are therefore inappropriate to use with this population.

#### Native signers.

Seventeen participants (11 women, 6 men) learned ASL from birth from deaf parents. The native signers served as the control group and show what argument ordering looks like given exposure to language from birth. The length of experience for the native signers was a mean of 30 years (SD=7.66), with their age matching their length of exposure. All of the native signers were bilingual in English (mean grade level reading = 7.9, SD=2.89). Two of the native signers had cochlear implants.

## Testing Procedure

**Figure 1.**
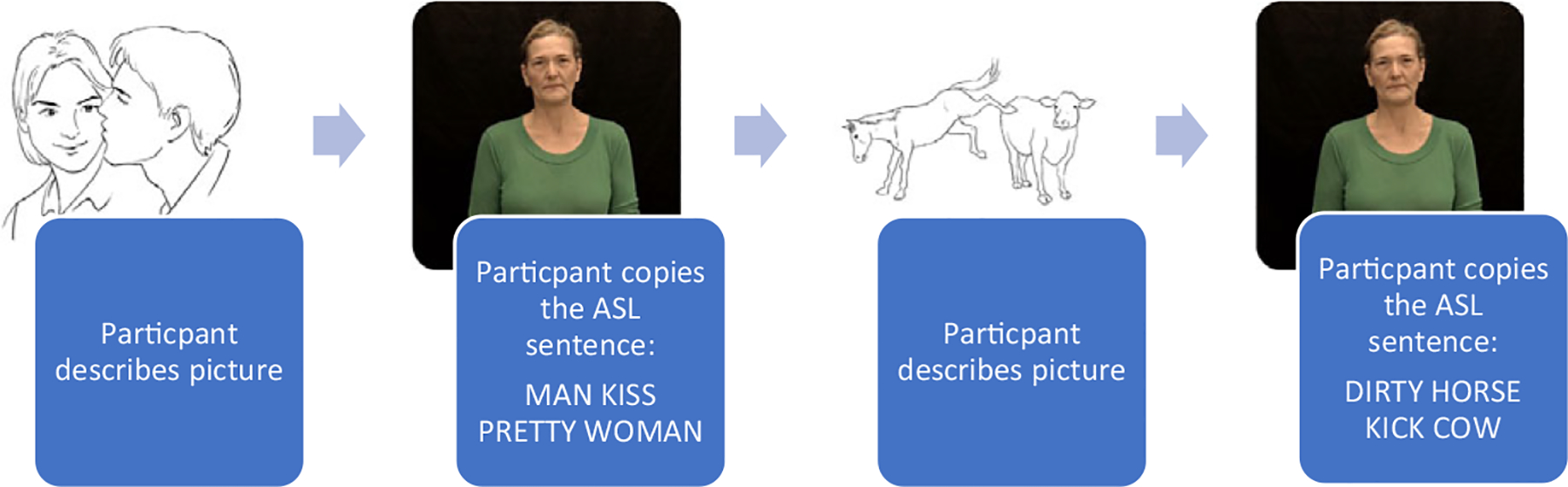
Sample of elicitation procedure for SVO syntactic block.

Participants were tested on a computer by either a deaf native or a hearing near-native ASL signer. As described above, the elicited production task was interleaved with an ASL elicited imitation task in an effort to prime sentence structure – that is, to provide multiple exposures to the target structure to encourage the participant to produce it. A total of 56 elicited production and 56 elicited imitation sentences were collected for each participant. During the instructions for each block, participants viewed a picture which was a line drawing paired with a video clip of a model sentence using the targeted structure. After instructions, participants saw a picture on a computer screen and described it – elicited production. Second, they saw a video of an ASL sentence using the target structure and repeated it – elicited imitation. Third, participants signed their description of the next picture designed to elicit the target structure and, fourth, repeated the stimulus ASL sentence describing the picture, again using the same target sentence but with different lexical items. This sequence of elicited production and imitation was repeated four times within each block. By the fourth elicited production trial in a block the participant had a total of four previous exposures to the syntactic structure in ASL (one model sentence in the instructions and three previous elicited imitation trials) along with an associated picture designed to depict the structure (Figure 1). Data for the current study were taken from the elicited production task. The order of the pictures within each block was randomized across participants. The order of blocks was also randomized.

## Coding Procedures

### Stimulus picture selection.

In order to select stimulus pictures depicting simple events with one or two arguments from the elicited production stimulus set, all of the pictures were coded according to the semantic relationships of the elements in each picture. For example, a picture showing a boy pulling a woman who was sitting in a wagon was coded for having an actor (transitive), patient, and instrument. A picture of a girl sleeping in bed was coded as having an actor (intransitive) and location. To test the hypothesis under investigation, we selected pictures consisting of three event types: intransitive, irreversible transitive, and reversible transitive. Hence, stimulus pictures depicting more than one event were excluded from the analysis, as were pictures illustrating reciprocal action. The signed productions were elicited by 20 pictured events: 8 intransitive, 12 transitive (9 reversible events and 3 irreversible). The uneven number of items per event type was due to the fact that larger study was designed to elicit a variety of syntactic structures in ASL so that event type was not controlled.

### Coding of participants’ signed productions.

The participants’ signed responses were coded by a native or near-native signer using ELAN. Each sign that the participant produced was glossed into English. In addition, nonmanual and prosodic marking was indicated in the gloss. After this initial coding, each sign in the response was additionally coded for whether it referred to the actor, patient, recipient, theme, or act of the pictured event. The sequence of signs was analyzed to discover ordering patterns as a function of event condition.

The participants’ response to each stimulus picture was then coded using the semantic relationships from the pictures, following Coppola (2002), Coppola and Newport (2005), and Neveu (2016). The act from the event was also coded. The coding categories included:

act - the action being performed
actor (tran) - the person performing the transitive action
actor (intr) - the person performing the intransitive action
patient - the person that is acted upon or manipulated
recipient - the person toward which something moves
location - the place towards which something or someone moves or is located
instrument - object used to complete the action
theme - inanimate object that is acted upon or manipulated
property - description of an object or person (e.g. tall, happy)

Some participants included more than one act in their responses. Therefore, acts were additionally coded for who was doing the action. For example, act(a) is an act completed by the actor from the picture while act(p) is an act completed by the patient from the picture. Because the events were shown in the stimuli, all responses were coded according to the roles of the arguments in the picture. We did not attempt to abstract the semantic roles from the signing of the participants but used the semantic roles from the event in the picture to analyze each participant’s signed response. In order to differentiate repetitions of the same act from additional acts, if a second or additional act was signed that was not a repetition, it was coded with a number such as act2 or act3. This coding can be seen in example (2) which was a response to a drawing of a woman kissing a man on the cheek and example (3) which was a response to a drawing of a man pulling a woman in a wagon. Note that in example (2), the second act is also marked as act(p) because it is completed by the patient from the picture. This will be discussed in the results section in more detail because it breaks the event into two clauses, one describing the action of the actor and one describing the action of the patient.

**Table:** 

(2)	GIRL	KISS	MAN	SMILE
	actor (tran)	act(a)	patient	act2(p)

**Table:** 

(3)	MAN	PULL	WOMAN	PULL
	actor (tran)	act(a)	patient	act (a)

Similarly, when a property was included in the response, it was coded as describing the actor or patient with the same notation ‘(a)’ or ‘(p)’ as acts. In example (4), the LL1 signer produced signs coded as properties for both the actor and the patient. The property referring to the woman who was the patient is marked with (p) and that referring to the actor is marked with (a).

**Table:** 

(4)	WOMAN	TALL,	BOY	YOUNG,	PULL
	patient	property (p),	actor (tran)	property (a)	act

In order to determine the order of arguments, each production was coded for the sequential order of the signs using the coded categories described above. Therefore, for intransitive sentences the coding was act-actor, actor-act, or unable to be determined (in cases where the act or actor was repeated such as act-actor-act). With transitive events, the order was coded over all three elements present in the picture (act, actor and patient or theme). When each element was signed one time in a sentence, this would lead to six possible orders. In addition, each production was coded for any omissions of arguments that were present in the picture and for repetitions of any arguments. The order of arguments was then determined for the elements explicitly signed. In example (5), which is a description of the picture of a girl eating a cookie, the signer omits the sign for cookie. Therefore, the order of arguments, for example (5), is actor-act (omit theme).

**Table:** 

(5)	GIRL	HAPPY	EAT
	actor	property	act

## Results

We first report the order of arguments the LL1 signers produced for each event type, intransitive, irreversible transitive, and reversible transitive with reference to the native signers. We then report the analyses of individual patterns. In total, the data analyzed were 478 spontaneously produced utterances by the 28 participants.

## Intransitive Events

The groups produced mostly actor-act order in response to drawings of intransitive events. Nonetheless, there was a statistically significant association between the signer group and the distribution of word orders, *X*^2^(1) = 29.72, p < 0.001. Effect size calculated using Cramer’s V, was 0.43, which is a medium to large effect. Specifically, all the native signers produced only the argument order of actor-act. The LL1 signers also tended to produce this order (68.75% of all productions) but they also produced other orders, as shown in Table 2. These other orders included few (2.5%) act-actor. The remainder of the productions either repeated or omitted the actor or act so that they could not be reliably judged as either actor-act or act-actor.

## Transitive Irreversible Events

For transitive events which were not reversible, there was again a significant association between the signer group and the distribution of word orders, *X*^2^(3) = 12.63, p = 0.006. The effect size calculated using Cramer’s V was 0.5 and is a large effect, indicating a strong association between group and ordering patterns. These events all included an animate actor and an inanimate theme. The most commonly produced pattern for both groups was actor-act (omit theme), as in example (5), repeated from above, given by a LL1 participant as a response to a drawing of a girl eating a cookie.

**Table:** 

(5)	GIRL	HAPPY	EAT
	actor	property	act

The other order produced by native signers was actor-act- theme. While this order was produced by the LL1 group, it was not the second most common order, unlike for the native signers.

The second most common order of arguments for transitive irreversible events among the LL1 group was actor-theme-act, which accounted for 20.7% of all responses, but was never produced by native signers. Other orders produced by LL1 signers, but not by native signers, included theme-act-actor; theme-act (omit actor); multiple clauses, and indeterminate. The rate of production of argument orders is shown in Table 3.

Thus, when native signers produced overt signs for all of the arguments in irreversible transitive events, they only produced actor-act-theme order. However, when LL1 signers produced overt signs for all arguments in the event, they produced a variety of orders of arguments. This was not only true for group level analysis: individual participants were not consistent in their argument ordering. Examples (6) and (7) were both produced by the same LL1 participant as responses to drawings of a girl eating a cookie and a boy reading a book.

**Table:** 

(6)	COOKIE	EAT	GIRL
	theme	act	actor

**Table:** 

(7)	BOY	READ	BOOK
	actor	act	theme

Thus, when only one argument was produced by the native signers and LL1 signers, they produced the order actor-act, as in intransitive events. However, when all arguments of the event were produced, the native signers all produced the same order, actor-act-theme, while the LL1 group used a variety of argument orders.

## Transitive Reversible Events

For reversible transitive events, there was again a significant association between group and argument ordering distribution *X*^2^(3) = 28.07, p < 0.001. Effect size calculated using Cramer’s V was 0.34, which is a medium effect, indicating a moderate association between the variables. These pictured events all included two animate arguments (actor and patient). The most common ordering pattern for both groups was actor-act-patient as seen in example (8), which was given as a response to a picture of a man kissing a woman on the cheek.

**Table:** 

(8)	MAN	KISS	WOMAN
	actor (tran)	act (a)	patient

This order of arguments aligns with the basic SVO word order of ASL and with the order used by native signers for irreversible events when both arguments were produced (actor-act- theme). Interestingly, neither native signers nor LL1 signers tended to drop the patient in these events although both groups tended to drop the theme in irreversible events. The full list of argument orders for transitive reversible events is found in Table 4.

The second most common production order for native signers was patient-actor-act, as seen in example (9). Topicalization of object in ASL produces O,SV word order with obligatory nonmanual markers including the prosodic break symbolized here by a comma (Liddell, 2003). When these patient-actor-act sentences were produced by LL1, they did not include the obligatory nonmanual or prosodic markers for topicalization.

**Table:** 

(9)	DOG	CAT	BITE
	patient	actor	act

The second most common order of arguments for the LL1 group was assigning an action to each animate argument. This pattern was seen in both groups, though more often in the LL1 signers. Since some participants did not produce consistent prosodic cues that could be used to determine clausal boundaries, responses were coded as having multiple clauses when an act was produced for both the actor and patient for the primary event pictured. As seen in example (10), the participant produced more than one clause with an act for both the actor and patient of the original event, which was a woman pulling a boy in a wagon.

**Table:** 

(10)	WOMAN	PULL	BOY	SIT	WALK	PARK
	actor	act (a)	patient	act2 (p)	act3 (a)	location

LL1 signers also produced other argument orders that were produced once or not at all by any native signer, including: actor-patient-act; patient-act (omit actor); patient-act-actor; actor-act (omit patient); and Indeterminate order.

To summarize, across all event types, there was a significant association between the order of arguments produced and the signer group. Although the LL1 signers did produce orders that were produced by the native signers, they did so at different rates. In addition, they produced argument orders that were not present in the signing of the native group. Both groups tended to produce actor-act order when only one argument was produced; however, the groups differed in their argument ordering when two arguments were produced. Native signers overwhelmingly produced actor-act-theme and actor-act-patient orders both of which correspond to ASL basic word order of SVO. By contrast, LL1 signers showed more variety in their argument ordering: the most common order for sentences containing both overt actor and overt theme was actor-theme-act order while the most common order for reversible events was actor-act-patient.

## Influence of Syntactic Context

While some similarity in argument ordering was observed in the native and LL1 signers, there were significant differences between the groups for the argument ordering as a function of event type. We next examined the factors that can potentially influence the variation in argument ordering. As previously explained, the data for the current analysis were taken from a larger study which was organized into syntactic blocks. For each block, participants saw a picture and described it. Next, they did an elicited imitation task, repeating a sentence about the picture they were seeing signed by a native signer. Recall that the pictures used here were selected based on analysis of the pictured elements to include only one event and one actor; therefore, the pictures appeared in a variety of syntactic blocks shown in Table 5. Thus, we initially looked at whether the participant’s production during the description task matched the syntactic block of the larger experiment, which was determined by the imitation task. For example, when the syntactic block was SVO order, the question is whether the participants also produced SVO order. Similarly, when the syntactic block was a topic-comment construction, did they produce O,SV order? To answer the question, we calculated Fisher’s exact test to determine if there was a significant association between group (native or LL1) and the number of signed productions which conformed or not to the structure of the syntactic block. As expected, the native signer group was significantly associated (Fisher’s exact test: p<0.001) with the production of utterances matching the syntactic block, producing matching sentence structures in 80.5% of all responses. By contrast, the LL1 group matched the syntactic structure in 31.8% of their responses, despite being exposed to the target structure four successive times.

## Individual Patterns

Due to the variation in argument orders produced by the participants for transitive events, we used Fisher’s exact test to determine if the variation in orders was equally distributed among the participants or if each participant tended to use some orders more than others. For each group and event type, matrices were created with each participant as a row and each argument ordering as a column. Cells were filled with the number of times the participant produced that argument ordering. For native signers, the results show independence between individual signers and argument ordering for irreversible (p=0.916) and reversible (p=0.08) transitive events. This suggests that the native signers were using grammatical structures similar to one another in response to the task because the variation in orders of arguments is equally distributed across participants. For LL1 signers, there was also evidence for equal distribution of argument orders for irreversible events (p=0.50). However, for reversible events, certain patterns were significantly more likely for some signers (p< 0.001). For example, half of the responses using actor-act-patient order came from just 3 of the 11 LL1 signers, while some LL1 signers never produced this order of arguments. Similarly, over half (52.4%) of productions of multiple clauses for reversible events were produced by 2 of the 11 signers, and this order was never used by 3 of the signers. These results indicate that the argument ordering of LL1 signers for single and dual argument events converge neither with one another nor with the ordering of arguments in basic clauses in the language they are learning, ASL.

## Discussion

To test the hypothesis that language acquisition begun after early childhood affects the development of basic clause structure, we analyzed the argument ordering patterns of 478 signed productions elicited by pictures of intransitive and irreversible and reversible transitive events by signers who first learned language after early childhood and those who learned from birth. The results provide strong evidence for the hypothesis. Signers with late acquisition used their own predominant orders regardless of the structure modeled in the context. Specifically, when only one argument in an event was signed, the LL1 signers tended to use actor-act order, as in intransitive events. However, when two arguments were mentioned (either actor and patient or actor and theme) for transitive events, they did not converge on an argument order and showed idiosyncratic ordering patterns. The results show, first, that early language input is required to develop the argument order rules of basic clause structure. Second, the results suggest that gesture, or homesign, patterns developed in early life persist in later language production when sign language learning begins after early childhood. Last, and importantly, the patterns found here for late language learners comport with those reported for emerging sign languages, as we discuss below.

For native signers, in both transitive event conditions when the patient/theme was expressed, the most common order was actor-act-patient/theme. These orders correspond to SVO, which is the basic word order of ASL. Recall also that SVO word order is required with plain verbs in the absence of other grammatical marking. We find here that native signers overwhelmingly follow this basic word order of ASL when describing pictures of single events with two arguments. Like other studies, these results indicate early language experience leads to the development of basic word order in sign languages (Novogrodsky et al., 2023). By contrast, the argument ordering of the LL1 signers varied as a function of transitive condition. Like native signers, their most commonly produced order for reversible events with two animate arguments was actor-act-patient; however, when both arguments were produced in irreversible transitive events, they did not converge on any single order of arguments. The order of arguments actor-theme- act, which was slightly more common than other orders used by LL1 signers, was never produced by any native signer. If the LL1 participants had learned the basic ASL word order pattern, then what was coded as patient and theme would occur in the same location in the sequencing of the grammatical object. Some LL1 signers showed actor-theme- act orders in irreversible scenarios. Although this pattern was not observed among the native signers, it does occur in some sign languages, especially for irreversible events with inanimate objects (Meir et al., 2017; Napoli & Sutton-Spence, 2014).

Deaf individuals who communicate with a gesture system within the family, often referred to as homesigners, show ordering patterns in their productive expressions. Goldin-Meadow and colleagues examined the homesign systems of young children both in the United States and Taiwan. They found that the children performed similarly in their two-sign productions with respect to whether they gestured the actor or patient arguments along with the act (Goldin-Meadow & Feldman, 1977; Goldin-Meadow & Mylander, 1998). One adult homesigner in Peru showed remarkably similar argument ordering to the children in the Goldin-Meadow studies and produced primarily two-sign sentences (Neveu, 2016). However, in a study of three adolescent and adult homesigners in Nicaragua where participants produced longer utterances, each participant disambiguated arguments through their own idiosyncratic order which did not match the orders in the other homesign studies (Coppola, 2002). Furthermore, the argument ordering in transitive events for all three homesigners differed depending on whether the object was animate (patient) or inanimate (theme). Specifically, one homesigner produced SOV for animate objects and SVO for inanimate objects while a second homesigner showed the opposite pattern of SVO for animate objects and SOV for inanimate objects, showing that they were sensitive to the animacy of arguments but did not converge on a single ordering of these arguments. Consistent with these findings, the productions of some of the LL1 signers showed sensitivity to argument animacy.

In homesign systems, argument ordering that is sensitive to animacy emerges early in childhood. The present results indicate that this pattern persists into adulthood when language acquisition begins after early childhood. That is, signers born deaf who grew up in an early environment with little perceptible language appear to retain some measure of idiosyncratic argument ordering from early childhood sensitive to the animacy of the arguments. One central question we cannot answer is whether the ordering patterns observed here were indeed used in the homesign systems of the present participants prior to learning ASL. Although nine of the eleven LL1 participants reported using gesture with their hearing families prior to learning ASL, we know nothing about whether and how it was structured, which is key to answering the question (Koulidobrova & Pichler, 2021).

The results also showed a tendency to use multiple clauses to describe transitive reversible events. Recall that in these cases the LL1 signers produced an action for each animate argument. Example (10) from above is repeated here.

**Table:** 

(10)	WOMAN	PULL	BOY	SIT	WALK	PARK
	actor	act (a)	patient	act2 (p)	act3 (a)	location

The tendency to produce only one animate argument per verb has also been observed in emerging sign languages. Emerging sign languages are young languages that have evolved among groups of deaf individuals and create a window through which we can observe how grammatical structures are formed over communicative time. De novo language formation has been described in situations of geographically isolated villages with a high incidence of deafness (Aronoff et al., 2008; de Vos, 2015; Ergin et al., 2018; Le Guen et al., 2020; Stamp & Sandler, 2021; Yano & Matsuoka, 2018) and in educational settings (Flaherty et al., 2014; Kocab et al., 2023; Senghas, 2003; Senghas & Coppola, 2001). The grammatical structure in language production of members of these signing communities is analyzed as a function of generations or cohorts of signers, with each generational wave being exposed to more language structure than the previous one. The first cohort are deaf individuals who began communicating without any sign language influence in the environment of their early childhood.

The first three cohorts of signers in Nicaraguan Sign Language (LSN) tended to produce multiple clauses with only one animate argument per verb when describing reversible scenarios (Flaherty, 2014; Flaherty et al., 2014). Like the present LL1 signers, the LSN signers were not exposed to accessible language from birth. Instead, they were able to communicate via sign only upon their entrance to the deaf school. Using multiple clauses with only one animate argument per verb has also been reported for the second generations of signers in two village sign languages: Al-Sayyid Bedouin Sign Language (ABSL) (Aronoff et al., 2008) and Central Taurus Sign Language (CTSL) (Ergin et al., 2018). Disambiguating arguments in reversible transitive events by assigning a separate action to both the actor and the patient argument appears to be a precursor to the emergence of the abstract ordering rules required by reversible transitive events.

Other studies have posited explanations of LL1 performance on sign language tasks that parallel the present findings. In a study of age of acquisition effects on deaf signers of Austrian Sign Language, Österreichische Gebärdensprache, the authors argue that the differences between those who acquired sign language post puberty (ages analogous with our LL1 group) versus earlier in childhood is explained by a greater reliance on what they call more perceptual and semantic levels of linguistic processing by the LL1 group (Krebs et al., 2021). Relatedly, Napoli and Sutton-Spence suggest that LL1 signers remain sensitive to parts of the grammar that are motivated by visual pressures, but have trouble with aspects of grammar that are arbitrary and language-specific (Napoli & Sutton-Spence, 2014). More research is needed to investigate how various perceptual, semantic, and pragmatic resources are recruited by older individuals when they are learning language for the first time.

The current study has several limitations. The analyses were done on elicited picture descriptions of events that were sampled unequally by event type. Within the 12 transitive events, 9 were reversible and only 3 were irreversible. However, this was unavoidable given that the pictures were taken from a larger study designed around syntactic structure and not event structure. Furthermore, the stimuli used for this elicitation did not control for animacy conditions. All actors across conditions were animate. Irreversible and reversible transitive events differed in the animacy of the patient/theme. The role of animacy in argument ordering requires further research. Despite these limitations, the current study adds valuable information to our understanding of the effects of delayed language experience on the learning of basic clause structure.

## Conclusion

It is known that spoken language acquisition is affected by the timing of the learning over human development (Lenneberg, 1967). Research has shown that the same is true for sign language, especially for complex syntactic structures. From analyses of signed production data, we find here that the acquisition of basic clause structure is affected by language acquisition begun after early childhood. These results extend the findings of previous results indicating that the development of basic clause structure is negatively impacted by limited language input during early childhood (Cheng & Mayberry, 2020; Mayberry et al., 2023) and thus helps explain why complex structures are difficult to learn after early childhood.

## Figures and Tables

**Table 1.** Age and ASL experience of the groups, Mean (SD)

	F/n	Age of ASL exposure	Age	Years of ASL experience	Reading Grade
Group					
LL1	7/11	14.37 (3.7)	34.27 (11.6)	10.36 (3.1)	1.9 (0.6)
Native	11/17	Birth (0)	29.59 (7.6)	29.59 (7.6)	7.9 (2.8)

**Table 2.** Argument order for intransitive events, percent (count)

Argument order	Late L1 signers	Native signers
Actor-Act	68.75 (55)	100 (106)
Act-Actor	2.5 (2)	0 (0)
Indeterminate	28.9 (23)	0 (0)

**Table 3.** Argument orders for transitive irreversible events, percent (count)

Argument order	Late L1 signers	Native signers
Actor-Act (omit Theme)	41.4 (12)	61.9(13)
Actor-Theme-Act	20.7 (6)	0 (0)
Actor-Act-Theme	13.8 (4)	38.1(8)
Multiple Clause	6.9 (2)	0 (0)
Theme-Act-Actor	3.4 (1)	0 (0)
Theme-Act (omit Actor)	3.4 (1)	0 (0)
Indeterminate	10.3 (3)	0 (0)

**Table 4.** Argument order for transitive reversible events, percent (count)

Argument order	Late L1 signers	Native signers
Actor-Act-Patient	44.7 (42)	59.6 (88)
Multiple Clauses	22.3 (21)	16.9 (25)
Patient-Actor-Act	10.6 (10)	21.0 (31)
Actor-Patient-Act	6.4 (6)	0.7 (1)
Actor-Act (omit Patient)	5.3 (5)	0.7 (1)
Patient-Act-Actor	2.1 (2)	0 (0)
Patient-Act (omit Actor)	2.1 (2)	0.7 (1)
Indeterminate	6.4 (6)	0.7 (1)

**Table 5.** Number of pictures from various syntactic blocks

Syntactic block	# Intransitive stimuli	# Transitive stimuli
SV	4	0
SVO	0	3
Topic-Comment	0	3
Modal/Aspectual Verb	1	2
Negation	1	2
Wh Question	2	2
Total	8	12
